# High Repetition Frequency Solid-State Green Laser with Large Stable Area for Water Jet Guided

**DOI:** 10.3390/mi14122231

**Published:** 2023-12-12

**Authors:** Ji Wang, Wenwu Zhang

**Affiliations:** 1 Ningbo Institute of Materials Technology & Engineering, Chinese Academy of Sciences, Ningbo 315201, China; zhangwenwu@nimte.ac.cn; 2 Zhejiang Key Laboratory of Aero Engine Extreme Manufacturing Technology, Ningbo 315201, China; 3 University of Chinese Academy of Sciences, Beijing 101408, China

**Keywords:** high stable, stable zone, water jet guided laser, low damage machining, deep cutting, green laser, high repetition frequency

## Abstract

This paper presents the design and experimental results of a long cavity length Nd:YAG laser with a large stable zone for water jet-guided laser (WJGL) applications. The design is based on the light transmission matrix and resonator stability conditions, aiming to achieve a large stable zone and a short cut-off thermal focal length (CTFL). A folded concave resonator is researched to enhance the cavity length, and the influence of the tunable cavity arm length on the oscillating beam in the resonator and in the YAG crystal is theoretically studied. Moreover, the effects of the output mirror curvature and the cavity arm length on the range of the stable area and the cut-off thermal focal length are also investigated. Experimental results show that a stable green laser output is obtained after second harmonic generation (SHG) with a pulse width ranging from 43 to 143 ns within the laser operating frequency range of 5–20 kHz. At an operation frequency of 10 kHz, the output power is 21.33 W, and the instability of the output power within 400 min is 0.88%. The laser source achieves a maximum power of 25.7 W at 20 kHz, and the maximum single pulse energy reaches 2.7 mJ at 6 kHz. Finally, this is used as the laser source to couple with a water jet with a diameter of 100 microns, achieving a lossless water conductivity transmission over 60 mm length. These results demonstrate the suitability of the designed laser source for WJGL technology research. In precision machining applications, this technology exhibits processing advantages of low thermal damage (~2 μm) and large depth (>10 mm), for 7075 aluminum alloy.

## 1. Introduction

A water jet-guided laser (WJGL) is a composite processing technology that has attracted the attention of scholars due to its features of minimal thermal damage, high precision, and powerful processing energy [1,2,3]. WJGL technology opens up new applications and possibilities for various fields, including precision machining, medical surgery, and aerospace engineering. In the field of precision machining, WJGL technology can offer precise and efficient cutting capabilities. The combination of a high-power laser beam and a micro-water jet enables the cutting with the advantage of high precision and minimal heat-affected zones. This technology has the potential to revolutionize industries such as jewelry [4], automotive [5], electronics [6], and aerospace [7,8], where precision cutting is crucial for manufacturing components. For example, in the aerospace industry, WJGL technology will be utilized for various purposes such as composite cutting [9,10] and turbine blades shaping [11].

In order to address the process challenges of large depth and quality consistency in water-guided machining, it is necessary to simultaneously address the issues regarding the stability, high power or high energy of lasers. The WJGL technology was invented by Synova Company [12]. At present, laser light sources used for water-guiding applications internationally generally have the following characteristics: (1) green light band, (2) laser operating frequency ranges from several thousand hertz to several tens of kilohertz, and (3) the output pulse width is in the order of hundreds of nanoseconds or microseconds. For example, Levent et al. [13] used a 25 W, 10 kHz, 200 ns, 532 nm laser to introduce a jet with a diameter of 150 microns for drilling chromium nickel iron alloy. Zhang et al. [14] conducted experimental research on carbon fiber composite cutting using a WJGL of 30 W, 40 kHz, 532 nm and a diameter of 100 microns. Qiao et al. [15] used 25 W, 300 ns, 20–120 kHz, 532 nm laser coupling with a jet diameter of 60 microns for research on cutting single-crystal silicon. Yang et al. [16] carried out processing research on a ceramic matrix composite through a 25 W, 20 ns, 30 kHz, 532 nm laser coupling with a jet of a diameter of 100 microns.

Considering that water-guided applications require efficient coupling with micron-scale water jets, there is a high demand for beam quality. Therefore, how to balance the output of high-power and high-beam-quality and ensure that the stability of the resonator does not decrease is the focus of research on water-guided laser light sources. Nd:YAG crystals have a high-efficiency four-level structure, but thermal effects limit the further improvement of the average power or energy of Nd:YAG lasers, and they also affect the stability and beam quality. The control of the stable region state of the resonator mainly lies in researching the thermal effect of laser crystals [17,18,19]. Sundar et al. [20] reduced the thermal lens effect and improved the output beam quality factor by rotating the pump LDs angle. Wang et al. [21] reported the research results of thermal-induced wavefront aberrations in liquid-cooled Nd:YAG thin plate lasers, achieving mutual compensation for wavefront differences. Wang et al. [22,23] develop a quasi-steady-state thermal model to analyze transient thermal effects in Nd:YAG laser crystal under a quasi-continuous laser-diode (LD) end pumping.

Here, based on the stable cavity design method, this paper reports a stable concave oscillating cavity by tuning the cavity length and adjusting mirror curvature to control the beam quality(M^2^~1.34). The effects of cavity arm length and cavity mirror curvature on the oscillating beam, stable region range, and cut-off thermal focal length were studied. A highly stable green laser with hundreds of nanosecond pulse widths was obtained at an operating frequency range of 5–20 kHz; the stability is 0.88% within 400 min. The maximum power and pulse energy was 25.7 W, 2.7 mJ respectively. The comprehensive indicators meet the requirements for light sources in the research of WJGL technology. Coupled with a micro-water jet of 100 μm diameter, several tens of millimeters of lossless WJGL transmission is achieved with a peak power density up to 0.742 GW/cm^2^. And a preliminary exploration was conducted on its application in precision machining.

## 2. Experimental Setup

**Figure 1:**
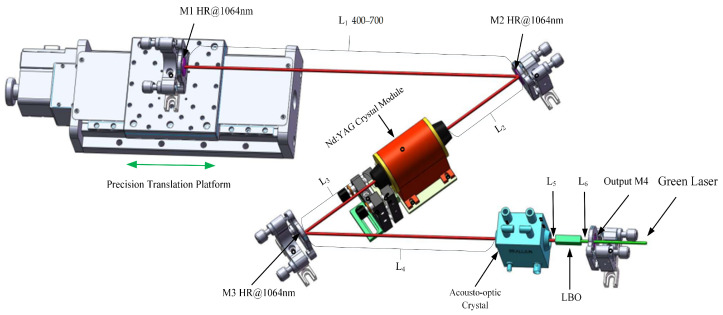
Tunable cavity length resonator device diagram.

The design of the stable spherical resonator is shown in Figure 1. The cavity mirrors are all concave mirrors, consisting of reflection mirrors M1, M2, and M3 and an output mirror, M4, with curvatures denoted as R_1_, R_2_, R_3_, and R_4_, respectively. Adopting semiconductor side pumping, the laser gain crystal is Nd:YAG, with a size of Φ2 × 60 mm and a doping concentration of 0.5 at.%, placed near the center of the cavity. The distance between cavity mirrors M1 and M2 is called the cavity arm L1. The distance between cavity mirror M2 and the laser crystal is denoted as L_2_, the distance between the laser crystal and cavity mirror M3 is denoted as L_3_, the distance between cavity mirror M3 and the acousto-optic crystal is denoted as L_4_, the distance between the acousto-optic crystal and the doubling crystal is denoted as L_5_, and the distance between the doubling crystal and cavity mirror M4 is denoted as L_6_. The cavity mirror M1 is fixed on a precision translation platform, model GCD-302001M (Manufactured by Daheng Optics Co., Ltd., from Beijing, China), with a repeated positioning accuracy of 0.05 mm, and the movement direction is consistent with the direction of cavity arm L_1_. Adopting intracavity frequency doubling, the frequency doubling crystal is LBO, placed near the output mirror M4, with a size of 3 mm × 3 mm × 15 mm; the θ angle is 90°, and the φ angle is 10.8°. One side near the acousto-optic crystal is coated with 1064 nm antireflection film (HR < 0.5%@1064 nm) and 532 nm high-reflection film (HR > 99.5%@532 nm), while the other side is coated with antireflection film (HR < 0.5%@1064 nm + 532 nm).

## 3. Theory

Due to the thermal lens effect, YAG crystals can be treated as a thick lens [24,25]; the thermal lens focal length noted as fth. For the resonator structure shown in Figure 1, according to the intracavity light transmission matrix, with the mirror M1 as the starting point, the paraxial round-trip matrix M of the base film Gaussian beam back and forth in the cavity is shown below:

(1) $$\begin{array}{l} M=\left[\begin{array}{cc} A & B\\ C & D \end{array}\right]=T_{12}\times T_{11}\times T_{10}\times T_{9}\times T_{8}\times T_{7}\times T_{6}\times T_{5}\times T_{4}\times T_{3}\times T_{2}\\ \times T_{1}\times T_{2}\times T_{3}\times T_{4}\times T_{5}\times T_{6}\times T_{7}\times T_{8}\times T_{9}\times T_{10}\times T_{11}\times T_{12}\\ =\left[\begin{array}{cc} 1 & L_{1}\\ 0 & 1 \end{array}\right]\times \left[\begin{array}{cc} 1 & 0\\ -\frac{2}{R_{2}} & 1 \end{array}\right]\times \left[\begin{array}{cc} 1 & L_{2}\\ 0 & 1 \end{array}\right]\times \left[\begin{array}{cc} 1 & \frac{L_{Y}}{n_{Y}}\\ -\frac{1}{fth} & 1 \end{array}\right]\times \left[\begin{array}{cc} 1 & L_{3}\\ 0 & 1 \end{array}\right]\times \left[\begin{array}{cc} 1 & 0\\ -\frac{2}{R_{3}} & 1 \end{array}\right]\\ \times \left[\begin{array}{cc} 1 & L_{4}\\ 0 & 1 \end{array}\right]\times \left[\begin{array}{cc} 1 & \frac{L_{Q}}{n_{Q}}\\ 0 & 1 \end{array}\right]\times \left[\begin{array}{cc} 1 & L_{5}\\ 0 & 1 \end{array}\right]\times \left[\begin{array}{cc} 1 & \frac{L_{B}}{n_{B}}\\ 0 & 1 \end{array}\right]\times \left[\begin{array}{cc} 1 & L_{6}\\ 0 & 1 \end{array}\right]\times \left[\begin{array}{cc} 1 & 0\\ -\frac{2}{R_{4}} & 1 \end{array}\right]\times \left[\begin{array}{cc} 1 & L_{6}\\ 0 & 1 \end{array}\right]\\ \times \left[\begin{array}{cc} 1 & \frac{L_{B}}{n_{B}}\\ 0 & 1 \end{array}\right]\times \left[\begin{array}{cc} 1 & L5\\ 0 & 1 \end{array}\right]\times \left[\begin{array}{cc} 1 & \frac{L_{Q}}{n_{Q}}\\ 0 & 1 \end{array}\right]\times \left[\begin{array}{cc} 1 & L_{4}\\ 0 & 1 \end{array}\right]\times \left[\begin{array}{cc} 1 & 0\\ -\frac{2}{R_{3}} & 1 \end{array}\right]\times \left[\begin{array}{cc} 1 & L_{3}\\ 0 & 1 \end{array}\right]\times \left[\begin{array}{cc} 1 & \frac{L_{Y}}{n_{Y}}\\ -\frac{1}{fth} & 1 \end{array}\right]\\ \times \left[\begin{array}{cc} 1 & L_{2}\\ 0 & 1 \end{array}\right]\times \left[\begin{array}{cc} 1 & 0\\ -\frac{2}{R_{2}} & 1 \end{array}\right]\times \left[\begin{array}{cc} 1 & L_{1}\\ 0 & 1 \end{array}\right] \end{array}$$

Here, in Equation (1), *L_Y_* is the length of the Nd:YAG crystal, *n_Y_* is the refractive index of Nd:YAG, *L_Q_* is the length of the acousto-optic crystal, *n_Q_* is the refractive index of the acousto-optic crystal, *L_B_* is the length of the LBO crystal, and *n_B_* is the refractive index of the LBO crystal.

The q parameter of intracavity self-reproducing Gaussian beam propagation is:

(2) $$\left\{\begin{array}{c} \frac{1}{q}=\frac{DA-A}{2B}\pm i\frac{\sqrt{1-{\left(D+A\right)}^{2}/4}}{B}\\ \frac{1}{q}=\frac{1}{r_{M}}-i\frac{\lambda }{\pi \omega_{M}{}^{2}} \end{array}\right.,$$

In Equation (2), *r_M_* is the curvature radius of the wave surface on the reference cavity mirror surface, and *ω_M_* is the size of the light spot on the reference cavity mirror surface. With the mirror M1 as the reference plane, after the transmission distance L′, the relationship between the propagation q parameter of the base film Gaussian beam on this plane (and the beam waist q_0_ parameter is as follows:

(3) $$q_{0}=q+L^{\prime },$$

Substituting Formulas (2) into (3) and combining it with the self-reproduction condition of the intracavity beam, the q_0_ parameter formula at the waist of the beam can be obtained as follows:

(4) $$\frac{1}{q_{0}}=\frac{1}{\frac{A-D}{2C}+L^{\prime }\pm \frac{\sqrt{1-{\left(\frac{A+D}{2}\right)}^{2}}}{C}},$$

Due to the fact that the wave surface at the waist of the intracavity transmission beam is a plane, the real part in Formula (4) should be 0. Formula (4) can be simplified, and the radius of the intracavity waist spot is shown below:

(5) $$\omega_{0}=\frac{\lambda \sqrt{1-{\left(\frac{A+D}{2}\right)}^{2}}}{\pi \left|C\right|},$$

Based on the size of the light spot on the mirror surface of the reference cavity or the beam waist in the resonator, the size of the oscillating light spot at any position in the resonator can be solved using Formula (3).

In the simulation calculation next, the curvature of R2 and R3 are both set as 500 mm, and the lengths from L_4_ to L_6_ are 195 mm, 5 mm, and 5 mm, respectively. The length of the acousto-optic crystal is 20 mm.

## 4. Simulation Calculations and Experimental Results

### 4.1. Influence of Cavity Length Tuning

**Figure 2:**
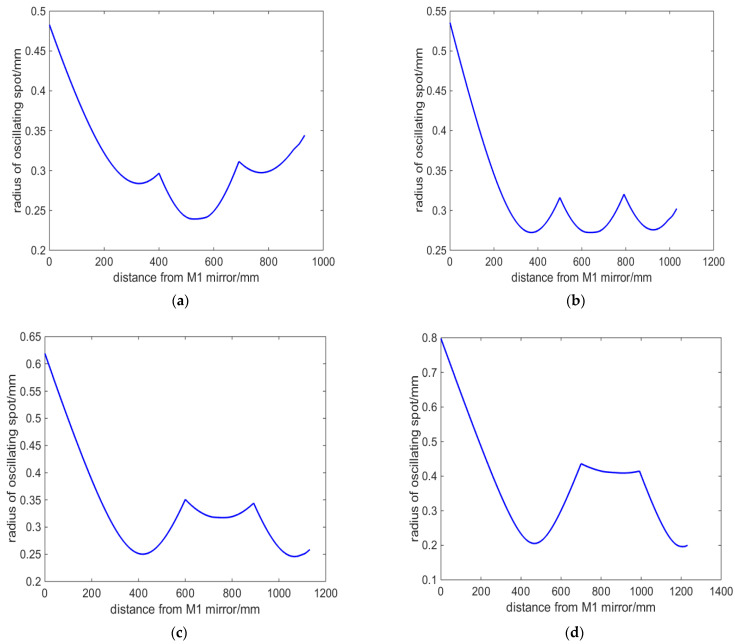
Simulation of intracavity oscillating spot with different cavity arm lengths: (**a**) L_1_ = 400 mm; (**b**) L_1_ = 500 mm; (**c**) L_1_ = 600 mm; (**d**) L_1_ = 700 mm.

According to the above formulas, we simulated and calculated the oscillating mode in the resonant cavity, and the results are shown in Figure 2. Figure 2a–d show the distribution of the oscillation beam radius at various locations in the resonator, corresponding to the cavity arm L_1_ lengths of 400 mm, 500 mm, 600 mm, and 700 mm, respectively. The vertical axis represents the oscillation beam radius, and the horizontal axis represents the positions with cavity mirror M1 as the origin. Corresponding to Figure 2a–d, the physical total length of the resonant cavity is 932 mm, 1032 mm, 1132 mm, and 1232 mm, respectively. It can be seen that, firstly, as the length of the cavity arm increases from 400 to 700 mm, even if the cavity length changes by 300 mm, the oscillation of the cavity beam can still be maintained stable without considering thermal lens. The radius of the oscillation beam waist spot in the cavity is 0.2392 mm, 0.2723 mm, 0.2458 mm, and 0.1960 mm, respectively. And as the cavity arm L_1_ increases, the waist position gradually moves toward the output mirror M4.

**Figure 3:**
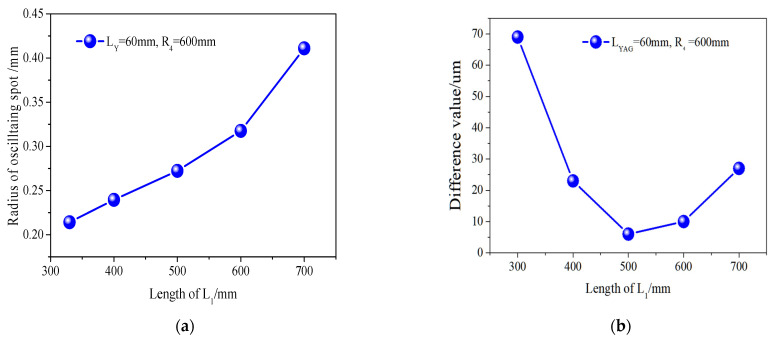
Oscillating spot in laser crystals with different cavity arm L_1_ lengths: (**a**) oscillating spot size at center of laser crystal; (**b**) difference value of oscillating light spot size in laser crystals.

According to the simulation calculation results in Figure 3, the laser crystal is placed at the center of mirror M2 and mirror M3, with lengths of 116 mm for both L_2_ and L_3_. We analyzed the oscillation spot situation in laser crystals with different cavity arm L1 lengths, and the results are shown in Figure 3. Figure 3a shows that when the length of the Nd:YAG crystal is 60 mm, the radius of the oscillating spot inside the crystal increases from 0.2142 to 0.4110 mm with the increase in L_1_. Reducing the size means that the volume of the intracavity mode is small, which affects the power output. Figure 3b shows the difference value between the maximum and minimum size of the oscillating spot in the Nd:YAG crystal. As L_1_ increases, the difference value decreases firstly and then increases. The overall difference value is less than 70 μm. When L_1_ is 500 mm, the minimum difference value is only 6 μm. The amplitude of change is approximately 3.1% of the size of the central oscillation spot.

### 4.2. Analysis of the Impact on the Stable Aera

**Figure 4:**
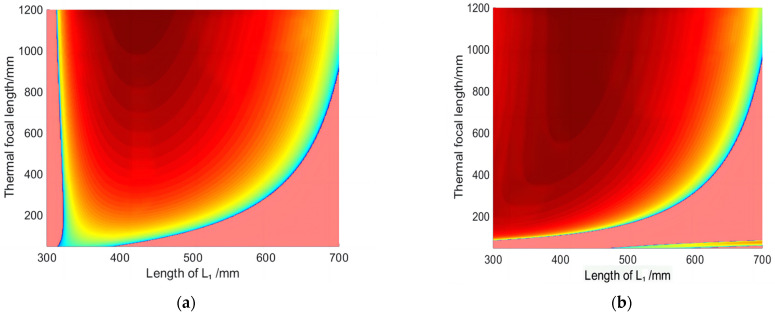

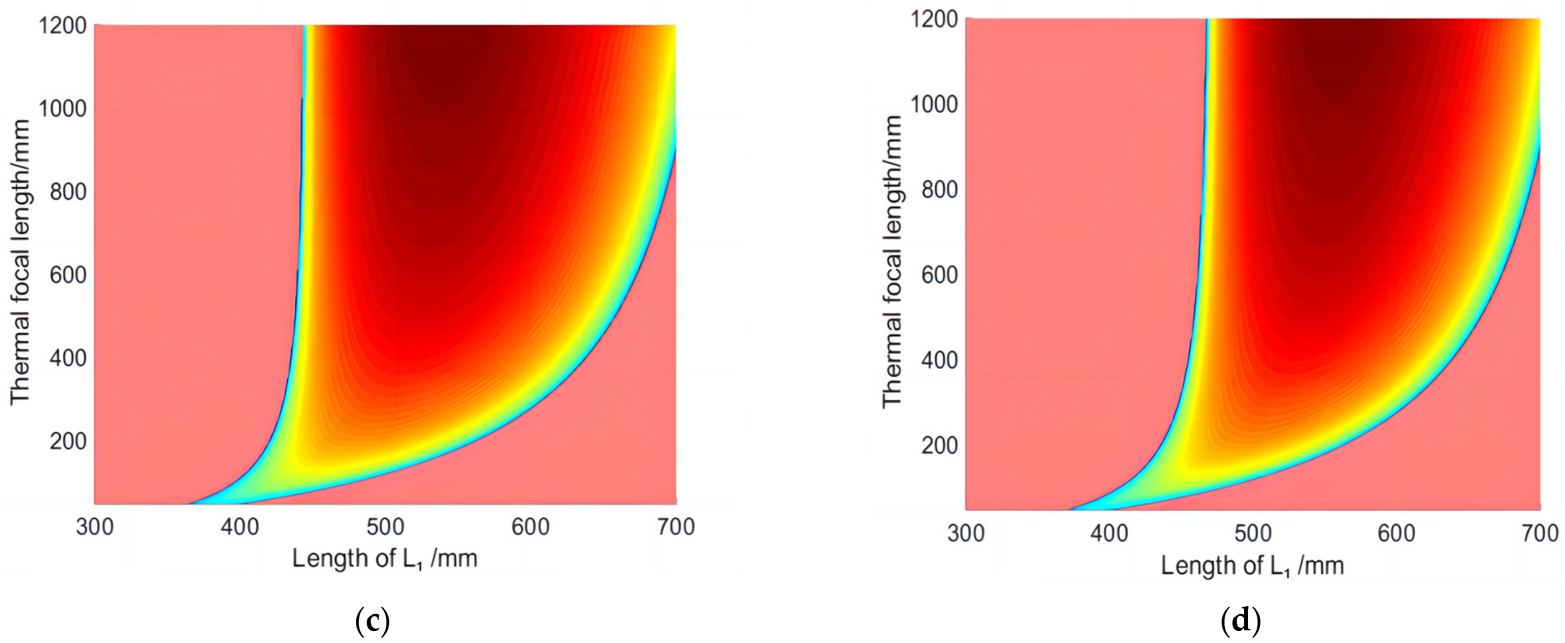
Stable region state of resonator: (**a**) R_4_ = 300 mm; (**b**) R_4_ = 600 mm; (**c**) R_4_ = 2000 mm; (**d**) R_4_ = 6000 mm.

Change the curvature R_4_ of cavity mirror M4 and perform stable zone calculations for different curvatures (R_4_ = 300 mm, 600 mm, 2000 mm, and 6000 mm). The corresponding results are shown in Figure 4a–d. The red area represents the stable zone range, the pink area represents the unstable zone range, the blue line represents the stable zone boundary, and the ordinate represents the thermal focal length. The thermal focal length at the boundary of the stable zone is the cut-off thermal focal length (CTFL). As a symbol of the stable state of the laser, the cut-off thermal focal length is the minimum thermal focal length that the laser meets in the stable zone state.

It is evident that the stable zone ranges in Figure 4a,b are larger than those in Figure 4c,d. In Figure 4c,d, when L_1_ is less than 500 mm, the cut-off thermal focal length is nearly infinite, much larger than 1200 mm, indicating that the resonant cavity is always in an unstable state. In addition, the stable zone ranges of Figure 4c is slightly larger than that of Figure 4d. Because, to be precise, in Figure 4c, when the cut-off thermal focal length reaches 1200 mm, the length of L_1_ is closer to 400 mm; whille in Figure 4d, the length of L_1_ is closer to 500 mm. It can be seen from the comparison that the curvature of the cavity mirror M4 should not be too large.

**Figure 5:**
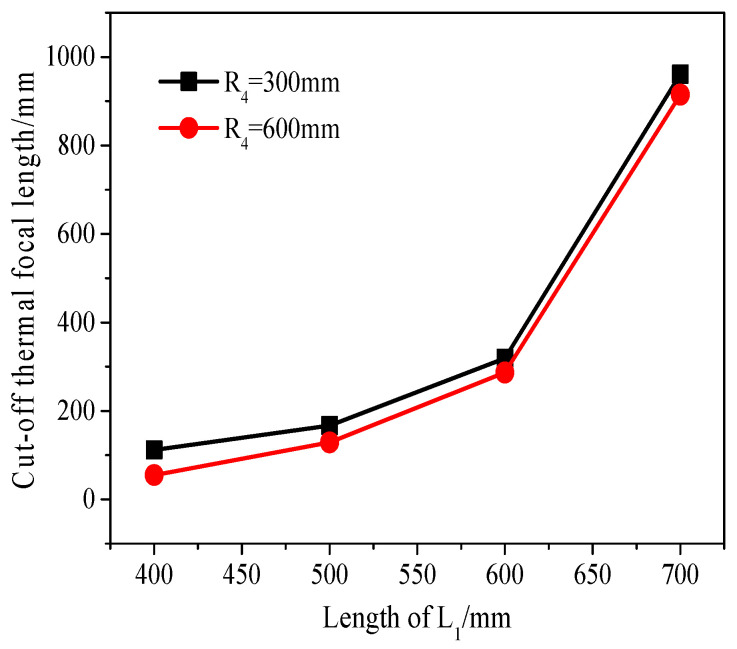
Relationship between length L_1_ and cut-off thermal focal length under different curvature R_4_.

Compare the cut-off thermal focal length under different cavity arms L_1_ in two modes (R_4_ = 300 mm, 600 mm). The results are shown in Figure 5, with the ordinate being CTFL. From Figure 5, it can be intuitively seen that when R_4_ is equal to 600 mm, as L_1_ changes from 400 to 700 mm, the length of CTFL is always shorter than the other curve. When R_4_ is 600 mm and L_1_ varies within 400–600 mm, the cut-off thermal focal length is 55 mm, 129 mm, and 287 mm, respectively, which reduces significantly compared to L_1_ at 700 mm.

**Figure 6:**
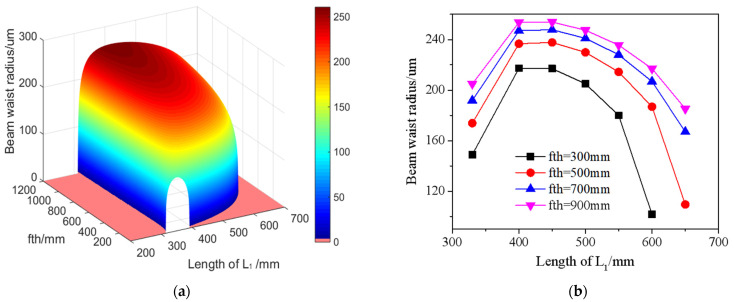
Beam waist radius at different thermal focal lengths in the stable area: (**a**) 3D view; (**b**) curve relationship at fth values of 300 mm, 500 mm, 700 mm, and 900 mm.

Figure 6 shows, at an R_4_ of 600 mm, the size state of the beam waist spot under different thermal focal lengths within the stable zone. The horizontal axis represents the positions. Figure 3a intuitively displays the relationship between the thermal focal length (fth), the beam waist radius in the resonant cavity, and cavity arm L_1_. As shown in Figure 6b, the waist beam spot size in the cavity shows a trend of firstly increasing and then decreasing consistently under the fth of 300 mm, 500 mm, 700 mm, and 900 mm. Under the same cavity arm L_1_ length, as the fth increases, the beam waist spot in the cavity becomes larger. When L_1_ is between 400 and 500 mm, the variation in the radius of the beam waist in the resonant cavity is relatively minimal, basically at a high-level value. It means that the stability of the output power will not be significantly fluctuated due to the changes in thermal focal length.

### 4.3. Experimental Results

**Figure 7:**
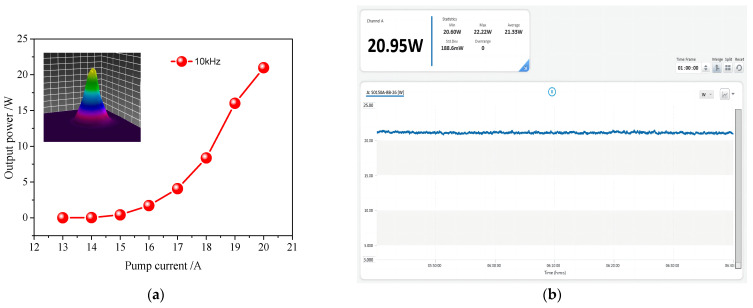
Power performance test results: (**a**) power curve at 10 kHz; (**b**) output power stability test.

We installed a flat concave mirror with a curvature of 600 mm on a precision motion table as the cavity mirror M1. Then, we set the curvature R_4_ of the cavity mirror M4 as 600 mm through the simulation calculation above. Through the control of a precise motion table, the length of cavity arm L_1_ is tuned as 500 mm, and the total physical length of the resonant cavity is 1032 mm. After LBO crystal phase matching frequency doubling, a green pulse light with an average power of 21.33 W is obtained at an operating frequency of 10 kHz. The average output laser power curve is shown in Figure 7. Figure 7a shows the curve variation of output green light power with a pump current, and Figure 7b shows the power stability monitoring result. As shown in Figure 7a, when the maximum pump current is reached, the average output power is 21.33 W. We conducted stability testing at maximum power, as shown in Figure 7b, and set the display cycle duration of the recording screen to 60 min. Within 400 min, the maximum output power was 22.22 W, the minimum output power was 20.60 W, the average output power was 21.33 W, and the root mean square value difference was 188.6 mW. The calculated root mean square (RMS) instability of the output power was 0.88%. To some extent, it reached industrial-grade stability performance. After transmitting a certain distance, the 3D shapes of the beam were observed on a light spot analyzer, as shown in the small image in the upper left corner of Figure 7a, with a roundness of about 92%. The model of the beam analyzer is Ophir Spiricon’s SP620U (Manufactured by Ophir Optronics Solutions Ltd., Jerusalem, Israel).

**Figure 8:**
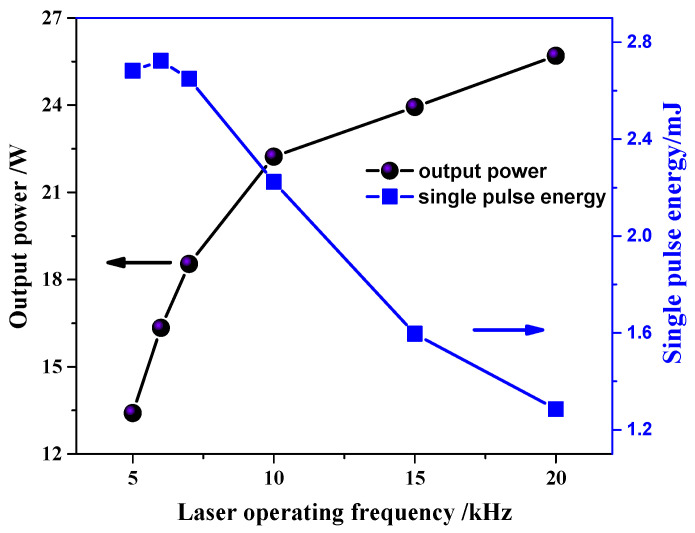
Output power and single pulse energy at different frequencies.

The maximum output power and single pulse energy curves at different frequencies are shown in Figure 8. The laser can maintain a stable working state within the operating frequency range of 5–20 kHz, and the maximum output power increases with the increasing of the operating frequency. The maximum output power at 20 kHz is 25.7 W. The growth rate of output power is slower than the increase rate of pulse operating frequency. The single pulse energy reaches its maximum of 2.72 mJ at approximately 6 kH. Within the range of 5–10 kHz, the single pulse energy remains above 2.0 mJ.

**Figure 9:**
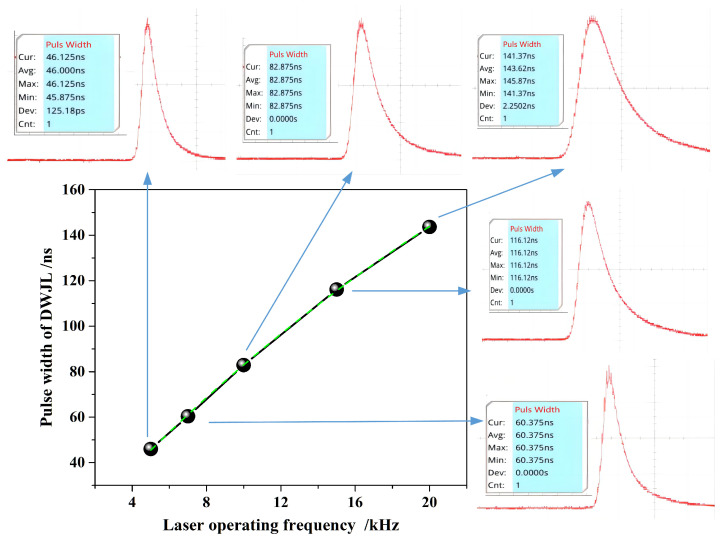
Pulse width at different operating frequencies.

The laser pulse width at different frequencies is shown in Figure 9. The pulse pictures at each operating frequency were collected using an oscilloscope (Manufactured by RIGOL Company, Suzhou, China, model: MSO5204), and the results are shown in Figure 9. It can be seen that as the frequency increases, the pulse width increases congruously. The average pulse width is 46.0 ns, 60.375 ns, 82.875 ns, 116.12 ns and 143.62 ns, corresponding to the frequency of 5 kHz, 7 kHz, 10 kHz, 15 kHZ and 20 kHz. We fit according to an exponential curve with a fitting function of:

(6) $$y=A1\cdot e^{-\frac{x}{t1}}+y_{0},$$

Here, A1 is −328.71092, t1 is 35.5073, and y_0_ is 331.0121. The fitting curve is shown by the green dashed line in Figure 9.

**Figure 10:**
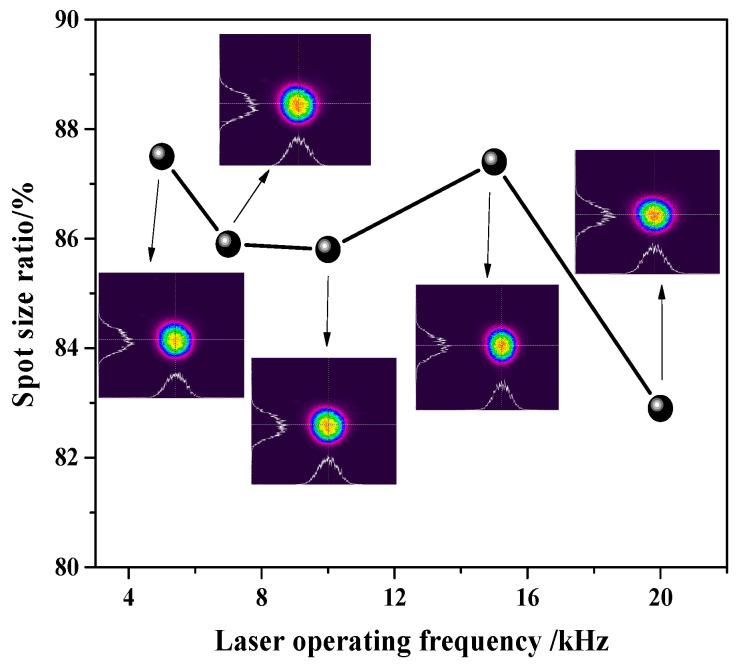
Spot morphology at different operating frequencies.

After the laser beam transfering through the reflector, wave plate, polarization crystal, and attenuation plates group, a beam quality analyzer is used to collect the morphology of the beam at maximum power with different frequencies, and the results are listed in Figure 10. The vertical axis in Figure 10 represents the ratio of the collected spot size in the y direction (short axis) to the size in the x direction (long axis). Considering that the parallelism of the optical axis of the added power attenuation optical module is not completely consistent, this to some extent affects the circularity of the light spot, which slightly decreases compared to the circularity of the three-dimensional light spot in Figure 7a. The long cavity of 1032 mm contributes to controlling the beam quality by mode selection. We measured the beam diameters at different distances from a lens at the maximum output power of 10 kHz. After calculating, the result of the beam quality M^2^ was approximately 1.34.

**Figure 11:**
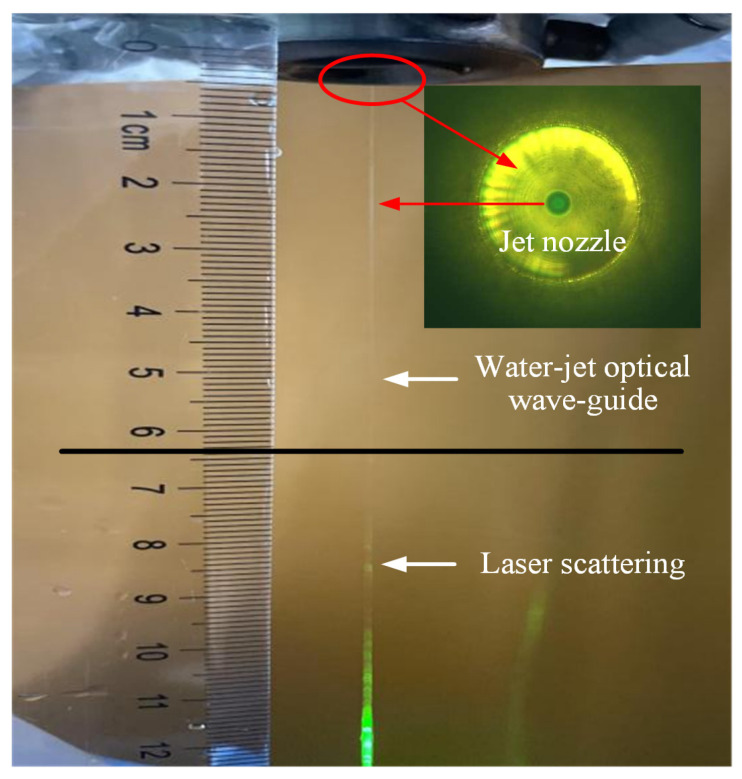
Water-guided laser based on the output laser pulse in this paper.

Finally, combined with the self-developed structure, the output pulse laser is tightly focused and coupled into the micro-water jet of 100 μm diameter, as shown in Figure 11. A smaller beam quality M^2^ factor is beneficial for obtaining smaller focused light spots after focusing. The small image in the upper right corner of Figure 11 shows the jet nozzle, and the small green light spot in the center of the nozzle is the coupled focused light spot. The plasma explosion threshold of laser in water is lower, and the water quality will further affect the explosion threshold. Plasma explosion will affect the total reflection, causing laser scattering in the water jet. In order to achieve lossless transmission, it is required that the laser operating frequency should not be too low and the single pulse energy should not be too large. From Figure 11, it can be seen that the laser undergoes total reflection in the water jet below the nozzle without any flash points of plasma explosion. After a certain distance of total reflection transmission, scattering phenomenon occurs. The total reflection transmission area has a length reaching 60 mm, and after exceeding the 65 mm area, scattering gradually occurs. This indicates that the high repetition rate of 5–20 kHz and high power green laser designed in this article is suitable for applying in the WJGL field, and it is especially suitable for the deep processing of materials. As the laser frequency increases, the peak power density of the WJGL gradually decreases. The maximum peak power density is 0.742 GW/cm^2^.

**Figure 12:**
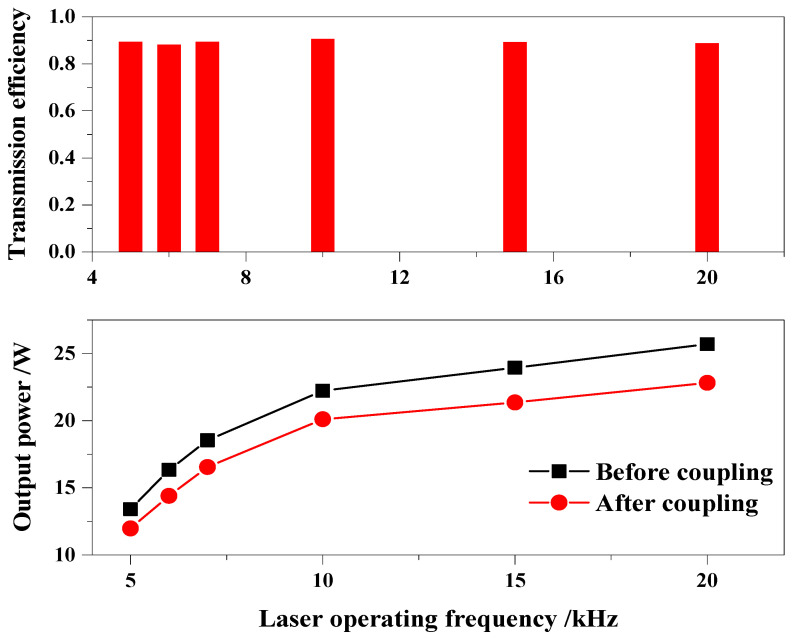
Transmission efficiency of laser power with water jet coupling transmission.

Furthermore, we calculated the transmission efficiency of a high-power laser coupled with that micro-water jet. When the highest output power was obtained at different operation frequencies (5–20 kHz), as shown in Figure 8, we measured the power values of the laser beam before and after coupling the water jet. The result is shown for Figure 12. As the frequency increases, the output laser power appears to have an increasing trend. We founded that the comprehensive transmission efficiency calculated is stable in the range of 88.2% to 90.5%, and the transmission efficiency was not affected by the operating frequency of the laser pulse. Considering various losses such as end-face coupling and water jet absorption, the transmission efficiency reaching 90% is an excellent result. This fully indicates that the laser beam obtained in this work has a widely operating frequency range.

### 4.4. Precision Machining Applications

**Figure 13:**
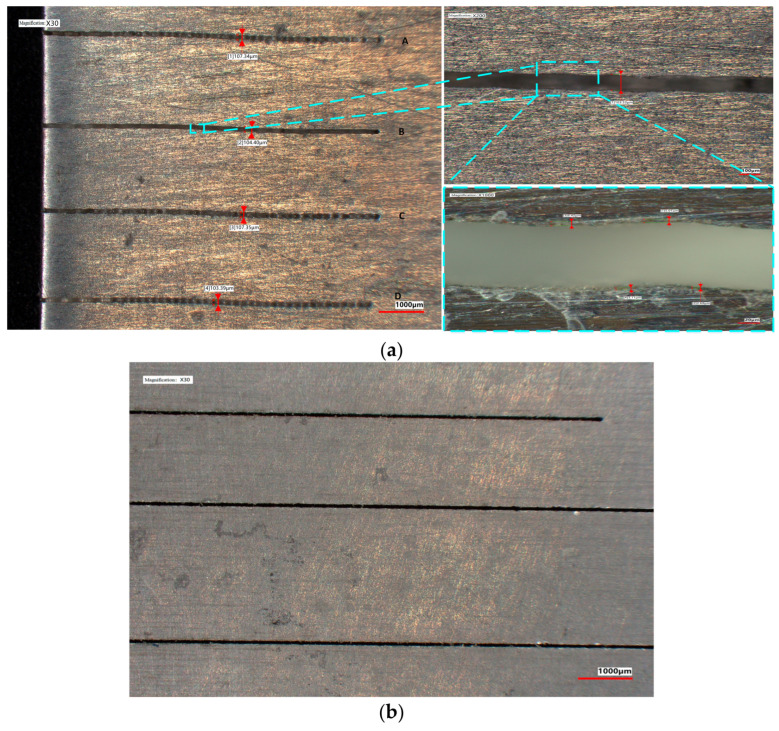
Low damage machining of 7075 aluminum alloy (with WJGL of ~21 W, 10 kHz). In (**a**) the process parameters for the machining grooves A–D are: groove A (single processing, feed rate of 1200 mm/s), groove B (reciprocating processing twice, feed rate of 200 mm/s), groove C (single processing, feed rate of 1200 mm/s), and groove D (reciprocating processing twice, feed rate of 1200 mm/s). (**b**) machinng three grooves after optimizing anti-shaking function of the motion with the same process parameters(reciprocating processing twice, feed rate of 200 mm/s).

The results in Figure 11 show that the green coupled focused light spot is significantly smaller than the 100 micron nozzle. Furthermore, it was found that in conjunction with parameters such as water flow pressure, the focused light spot can be coupled into a finer sized water jet. Then, we conducted precision machining application experiments for this laser. Using a ~21 W, 10 kHz laser pulse, 7075 aluminum alloy was processed to form machining grooves on the surface, as shown in Figure 13a (the figures were tested by KEYENCE’s ultra-depth of field microscope, lens model: VH-ZST). We marked the machining groove as A–D from top to bottom in Figure 13a, with specific process parameters as follows: groove A (single processing, feed rate of 1200 mm/s), groove B (reciprocating processing twice, feed rate of 200 mm/s), groove C (single processing, feed rate of 1200 mm/s), and groove D (reciprocating processing twice, feed rate of 1200 mm/s). Obviously, the feed speed is too fast, resulting in an excessive separation of laser pulses. With a 100 micron water jet, the processing width of grooves A to D is 107.34 μm, 103.40 μm, 107.34 μm, and 103.39 μm in sequence. We used a high-power microscope (lens magnification ×1000) to observe the thermal erosion marks on the edge of the processing grooves. No significant material discoloration or remelting phenomenon caused by thermal ablation was observed, confirming the low thermal damage characteristic of WJGL processing. The edge comprehensive damage diffusion area is approximately 2–7 μm, which was combined with thermal damage and impact damage. Referring to the process parameters used for machining groove B, the anti-shaking function of the motion axis was optimized and straight-lines machining was performed. The results are shown in Figure 13b.

**Figure 14:**
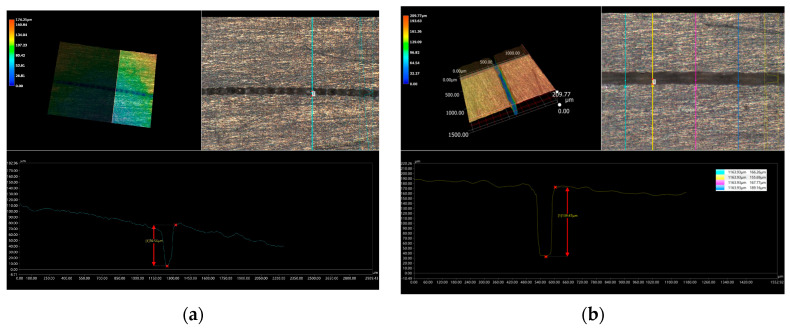
Comparison of three-dimensional morphology between once and twice processing (with WJGL of ~21 W, 10 kHz). (**a**) is the three-dimensional morphology of machining groove A in Figure 13a, (**b**) is the three-dimensional morphology of machining groove B in Figure 13a.

We measured the three-dimensional morphology of machining grooves A and B in Figure 13a, as given in Figure 14. The depth of processing once is 70.56 μm. Four different positions of groove B were measured, and the measured depth values of processing twice are 166.26 μm, 155.69 μm, 167.77 μm, and 189.16 μm, while the average depth is 169.72 μm. The depth of the two-processing operation reaches more than twice that of a single operation. Moreover, the perpendicularity of the grooves formed by twice processing is significantly better than that of a single process. The section of groove B in Figure 13b is concave at nearly 90°, whereas the section of groove A in Figure 13a is V-shaped with a noticeable taper. Due to that, via multiple processing, the removal effect can be better accumulated, and the taper of the groove section can be flattened layer by layer.

**Figure 15:**
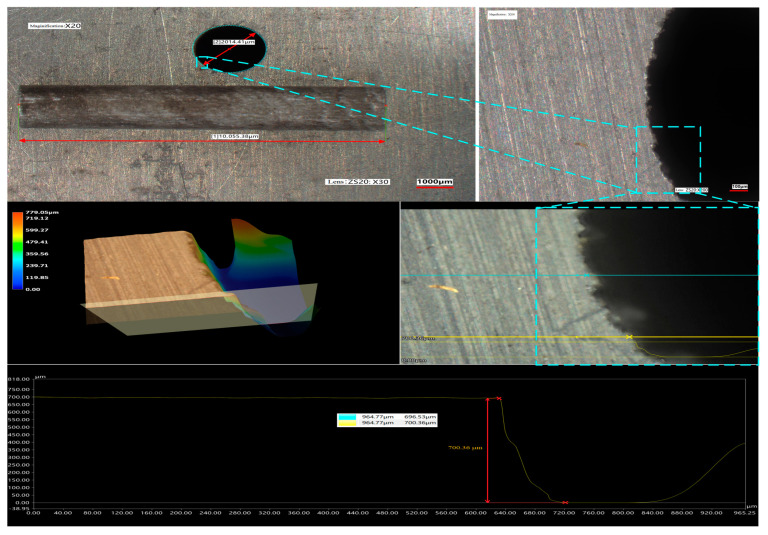
Cutting applications for large processing depth.

Furthermore, this WJGL is utilized for deep processing. Multiple cuts were made to achieve the cutting through of 7075 aluminum alloy sheet with a thickness of 10.05 mm (>10 mm). As shown in Figure 15, a through type circular hole was cut on the board by using a circular motion trajectory. The appearance of the dropped cylinder is in good condition, without fracture, and can match with the through-hole. The diameter of the circular through-hole is 2.014 mm, which is slightly larger than 2.0 mm. That is because for deep cutting, it is necessary to leave a small channel in the machining groove for water jet circulation so as to timely remove heat and material powder generated during processing. Similarly, no obvious discoloration or remelting caused by thermal erosion was observed around the machined edge of these grooves.

## 5. Discussion

Concave mirrors have a converging effect on the beam. Therefore, by using a four-mirror concave cavity, it is possible to obtain three converging oscillating beams in the resonant cavity. Adjusting the curvature of the cavity mirror can modify the size of the oscillating beam. From Figure 2a–d, it is found that the resonant cavity designed has an important characteristic. No matter how the cavity length changes, there is always a section of oscillating beam between the cavity mirrors M2 and M3, which is close to the parallel beam. By utilizing this characteristic, placing the laser crystal Nd:YAG at this position in the cavity can effectively balance the temperature field distribution inside the laser crystal. The result is that, as can be seen from Figure 3b, the minimum difference in the size of the oscillating spot within the laser crystal is only 6 μm. And the variation of L1 within the range of 400 to 600 mm has little impact on the mode of the oscillation spot in the resonant cavity, including the waist size and the difference sizes of the oscillation spots in the crystal.

**Figure 16:**
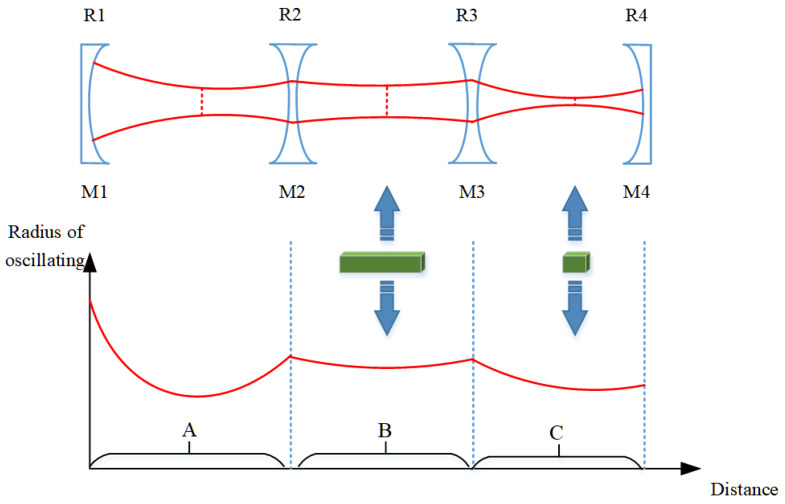
Schematic diagram of oscillating beam in concave mirror resonant cavity.

As shown in Figure 1, a foldable resonant cavity designed with a set of concave mirrors can be expanded into a linear shape. The equivalent resonant cavity is shown in Figure 16. The curvature on both sides of cavity mirror M2 is equal; similarly, the curvature on both sides of cavity mirror M3 is also equal. A concave mirror has the ability to concentrate light beams. Therefore, by using a four-mirror concave cavity, three converging oscillating light spots can be obtained in the resonant cavity. The resonant cavity is divided into three regions, labeled A, B, and C, where these three converging oscillating light spots are located, respectively. Region A corresponds to the region with a length of L1 from mirror M1 to mirror M2 in Figure 1, Region B corresponds to the region range from mirror M2 to mirror M3 in Figure 1, and Region C corresponds to the region range from mirror M3 to output mirror M4 in Figure 1. By referring to telescope theory, the size of the oscillating beam can be accurately changed by changing the curvature or spacing of the cavity mirror.

Firstly, the cavity mirrors M1 and M2 form a confocal cavity. The curvatures R_1_ and R_2_ can be adjusted to control the size of the converging beam in region A. The length L_1_ of the cavity arm is adjusted to ensure that the oscillating beam in region A converges at the focal point of the left surface of the cavity mirror M2. Then, it is important to ensure that the oscillating beam in region B gradually obtains a parallel light configuration. This configuration is suitable for placing large length laser gain crystals. This characteristic is illustrated in Figure 2a–d: regardless of changes in the cavity length, there is always an oscillating beam between the cavity mirrors M2 and M3 that closely resembles a parallel beam. By utilizing this characteristic, the laser crystal Nd:YAG can be effectively positioned at this location in the cavity to achieve a balanced temperature field distribution inside the laser crystal. Furthermore, Figure 3b shows that the oscillating spot size difference within the laser crystal is only 6 μm. The variation of L1 between 400 and 600 mm has minimal impact on the overall oscillation mode of the resonant cavity, consistently maintaining three-stage convergence regions. The parallel beams formed by region B converge again after passing through M3, creating a common focus within region C. The size of the oscillating spot can be quickly modified by adjusting the curvature of either the front or rear mirrors. For example, changing the curvature of the rear cavity mirror M4 can affect the beam in region C. According to the principles of telescopes, there is a specific numerical relationship between the beam focus in region C and the beam focus in region A, which naturally alters the size of the oscillating beam in region A. In this study, the position of the rear cavity mirror M4 is fixed, which is advantageous for use as an output mirror. Therefore, region C is designated as the placement area for Q-switching crystals and frequency-doubling crystals.

As the laser power increases, the thermal focal length within the crystal becomes shorter. The CTFL symbolizes the laser’s stable state and represents the minimum thermal focal length achievable in this state. To maintain the stable state of the resonant cavity and minimize the influence of the thermal lens effect, it is necessary to minimize the CTFL as much as possible. Considering all factors and referring to Figure 5 and Figure 6, the curvature R4 of the cavity mirror M4 is selected as 600 mm. L1 is determined to be 500 mm. Additionally, the CFTL, which meets the critical stability zone conditions, is as low as 129 mm. This exceeds the limit of the thermal focal length that laser crystals can reach when operating at high power, ensuring the long-term stability of the laser.

**Figure 17:**
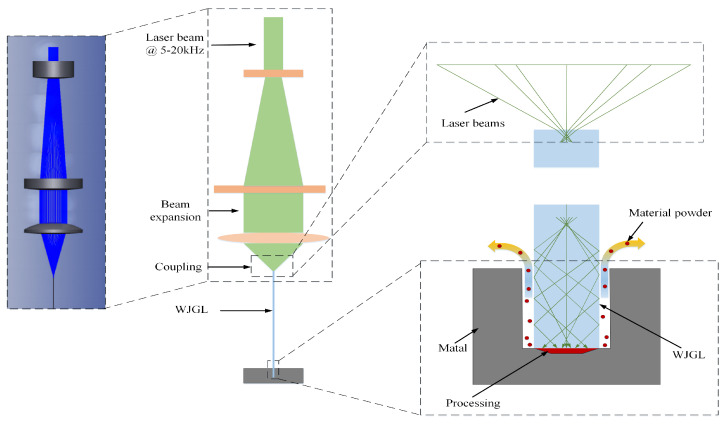
Schematic diagram of WJGL on beam modulation and deep processing.

It can be seen that the size difference between the coupled focused laser spot and the jet nozzle is only a few tens of micrometers from that shown in Figure 11. Especially, when further reducing the diameter of the water jet, if the stability of the focused laser spot under different output power conditions and long-term operation cannot be guaranteed, it is easy to cause fluctuations of the focusing spot morphology, leading to laser ablation of the nozzle and damage to the formation of the optical wave-guide. The high stability green laser with an operation range of 5–20 kHz designed in this study overcomes this difficulty. The muti-physical model includes the modulation process of the high-stability green laser beam and the interaction between the WJGL and the materials listed in Figure 17. The beam was expanded firstly, then focused by a lens, and finally coupled with a micro-water jet on the end face. While the laser incidence angle was kept below the critical angle, the beam will undergo total reflection within the jet; then, it will be transmitted onto the surface of the materials and begin material removal. Adjusting the beam diameter and lens focal length in this study ensures good coupling efficiency by modifying the incident angle. After the water jet optical wave guide reaches the material surface, the heat generated in the processing area is quickly carried away due to the high-speed scouring effect of the water flow in the groove gap, as shown in Figure 15. This plays a role in weakening thermal diffusion and thermal erosion. So, the processing with WJGL has low thermal damage, as given in Figure 13 and Figure 15.

For thin plate cutting, the depth of processing increases in direct proportion to the number of processing times and laser power. In the case of deep machining, the depth of machining follows a curve with a decreasing slope. As the depth of the machining groove continues to increase, two difficulties have been identified in engineering. Firstly, the removed materials form dust or plasma clusters, which fill the deep grooves and are difficult to sputter out with water flow, leading to the absorption and scattering of laser pulses, resulting in decreasing later processing ability. Secondly, as the groove deepens, the outward splashing water flow will interfere with the downward spraying laser water jet, affecting the timely removal of heat. Secondly, as the groove deepens, the outward splashing water jet will interfere with the downward spraying laser water jet, affecting the timely removal of heat. Therefore, it is necessary to fully consider the planning of processing paths in practical processing applications in order to facilitate the timely discharge of water flow in groove gap. This article has achieved low damage machining with a depth exceeding 10 mm, adopting a simple single-channel machining path. As shown in Figure 11, the length of the water jet optical wave-guide area for laser non-destructive transmission is far more than 10 mm. Based on in-depth exploration of process parameters and processing paths, it is believed that low loss processing depth can be greatly improved in the future. When we figure this out, it will be a significant moment.

Of course, there are differences in thermal damage thresholds and processing mechanisms for different materials. The laser for water-guided laser application in this study has the advantage of a wide frequency adjustable range. High operation frequency is associated with a high output power for a laser and low operation frequency is associated with a low output power for a laser but high single-pulse energy. For materials susceptible to thermal erosion or oxidation, such as metal and fiber composite materials, it may be advisable to choose a medium to low frequency range to prevent water jets from failing to remove the heat accumulated due to high-frequency pulses in time. The specific process parameters need to be optimized and explored, and detailed analysis and discussion will not be conducted in this paper. In order to improve the straightness of the processing edge, it is necessary to consider the overlap rate of the light spot before processing, as shown in Formula (7).

(7) $$\left(1-k\right)\times D=v\times f,$$

Here, the overlap rate of the light spot is k, the diameter of the water jet optical wave-guide is D, the feed rate is v, and the laser operation frequency is f.

## 6. Conclusions

In summary, based on long resonant cavity mode selection and stable zone control, this article achieves a high stability and high-power pulsed laser, which can be used for the long distance lossless transmission of WJGL. It provides strong technical support for the deep, efficient and high-quality processing of materials. Compared with the current process parameters of water-guided laser processing, this high repetition rate of the Nd:YAG green laser with a large stable range and high stability is a very suitable laser source.

In this paper, we designed a folded concave oscillating cavity, which has the characteristic of the tunable length of the cavity arm in the stable zone state. Using the ray transfer matrix method, the influence of the length of the tuning cavity arm L_1_ on the oscillating beam in the cavity and crystal is analyzed. Furthermore, the effects of the output mirror curvature and cavity arm L_1_ on the scope of the stable zone and the CTFL are studied, taking into account the thermal focal effect. Research results have shown the following:

(1) When R_4_ is 600 mm, the stable zone scope is sufficiently large. Meanwhile, when L_1_ varies between 400 and 600 mm, the change amplitude of the size of the beam waist in the resonator and the CTFL are both relatively low, which can effectively suppress the influence of thermal focal effect to stabilize the output power.
(2) According to the design, the total physical length of the resonant cavity is 1032 mm. In addition, 21.33 W of average power and 0.88% (RMS) of unstable value is obtained at a working frequency of 10 kHz within 400 min. Changing the pulse frequency to 5–20 kHz the resonator can still maintain stable operation with a maximum output power of 25.7 W and a maximum single pulse energy of 2.7 mJ.
(3) After experimental verification, the laser designed in this paper is perfectly coupled with a water jet of 100 microns diameter, achieving lossless transmission over 60 mm for a water jet-guided laser. In precision machining applications, this technology exhibits processing advantages of low thermal damage (~2 μm) and large processing depth (>10 mm) for 7075 aluminum alloy.

## Data Availability

The data that support the findings of this study are available from the corresponding author upon reasonable request.
